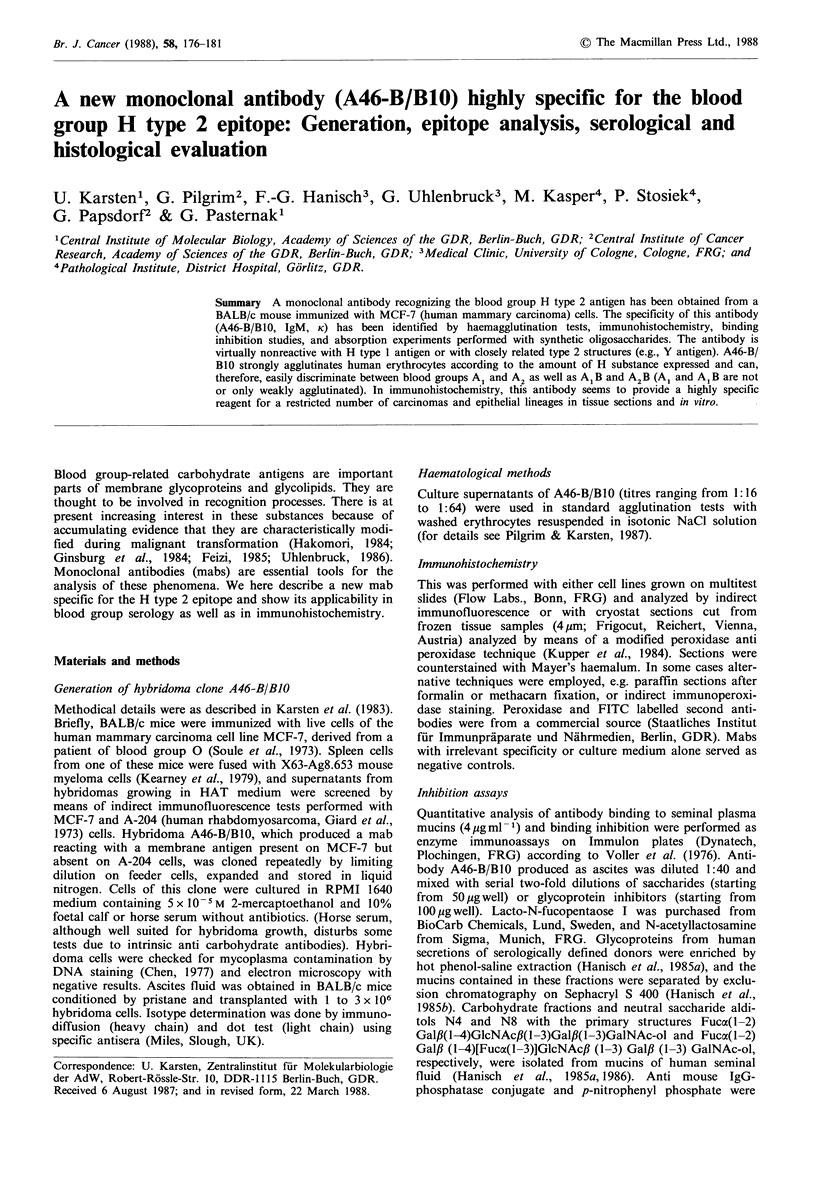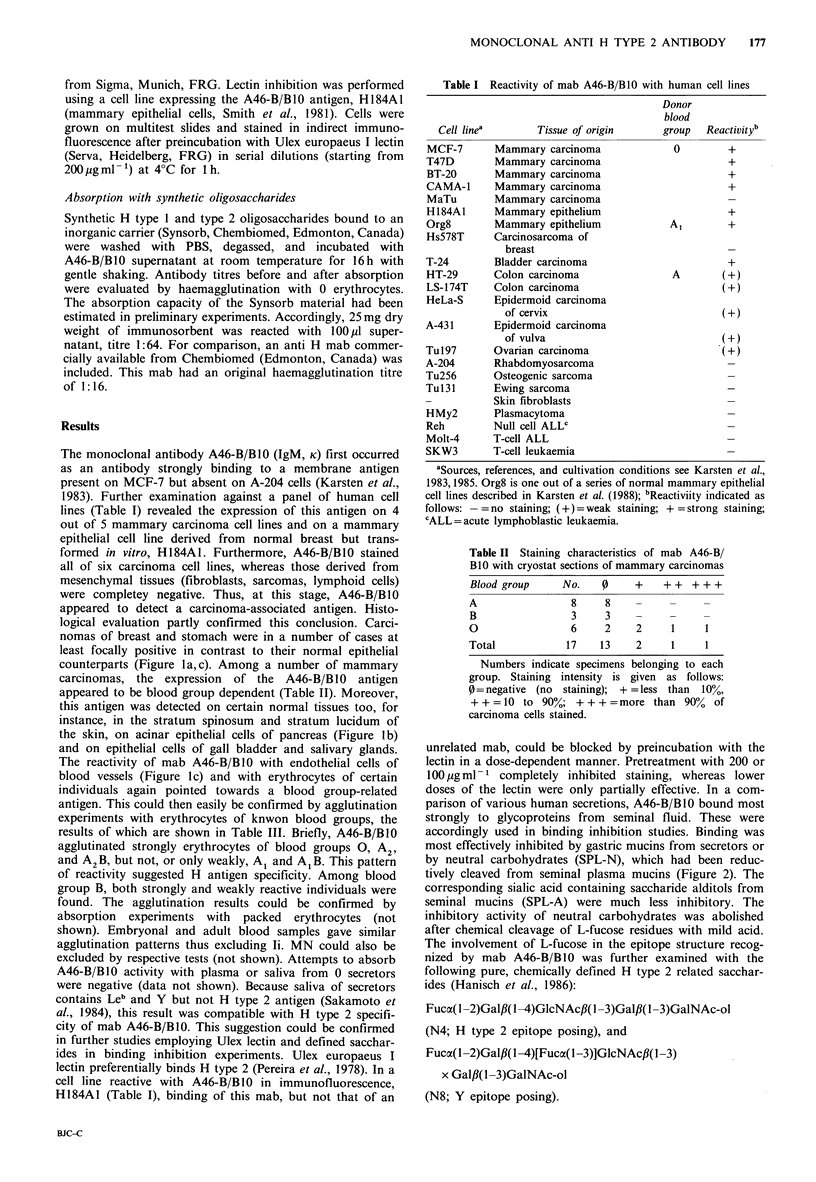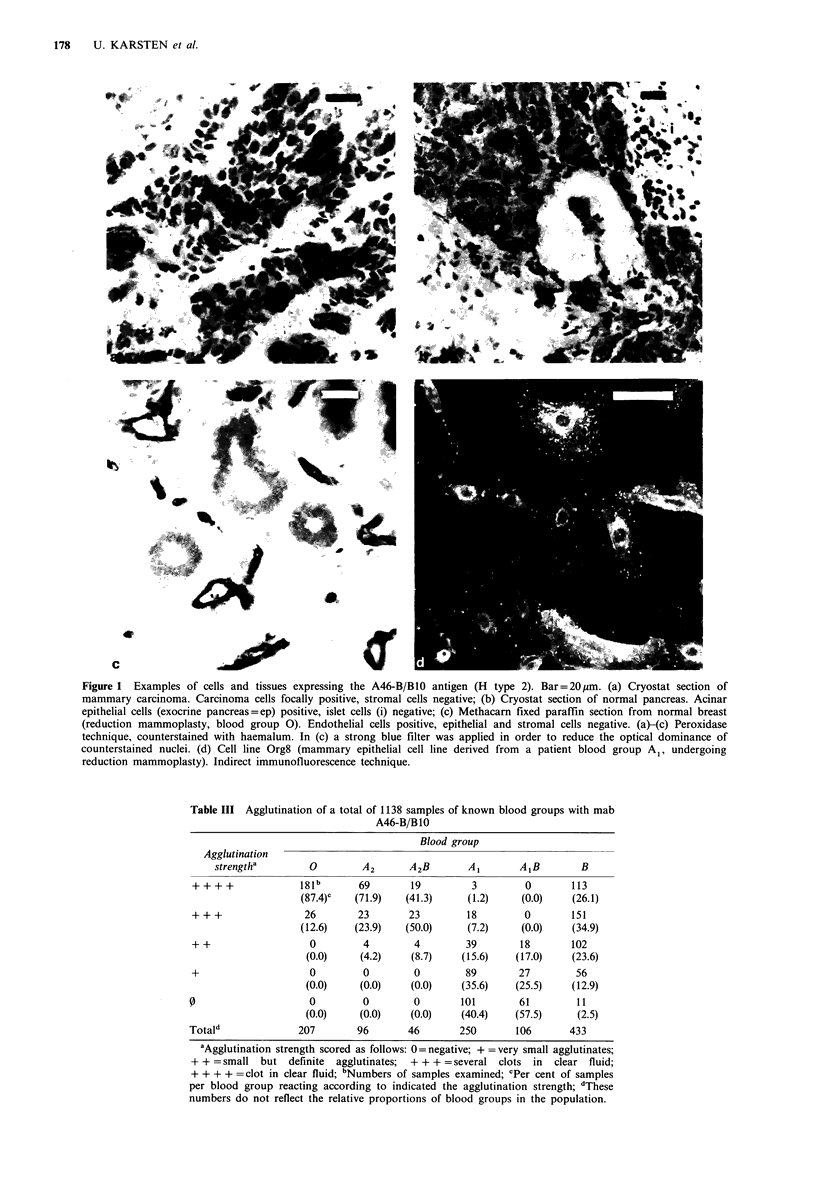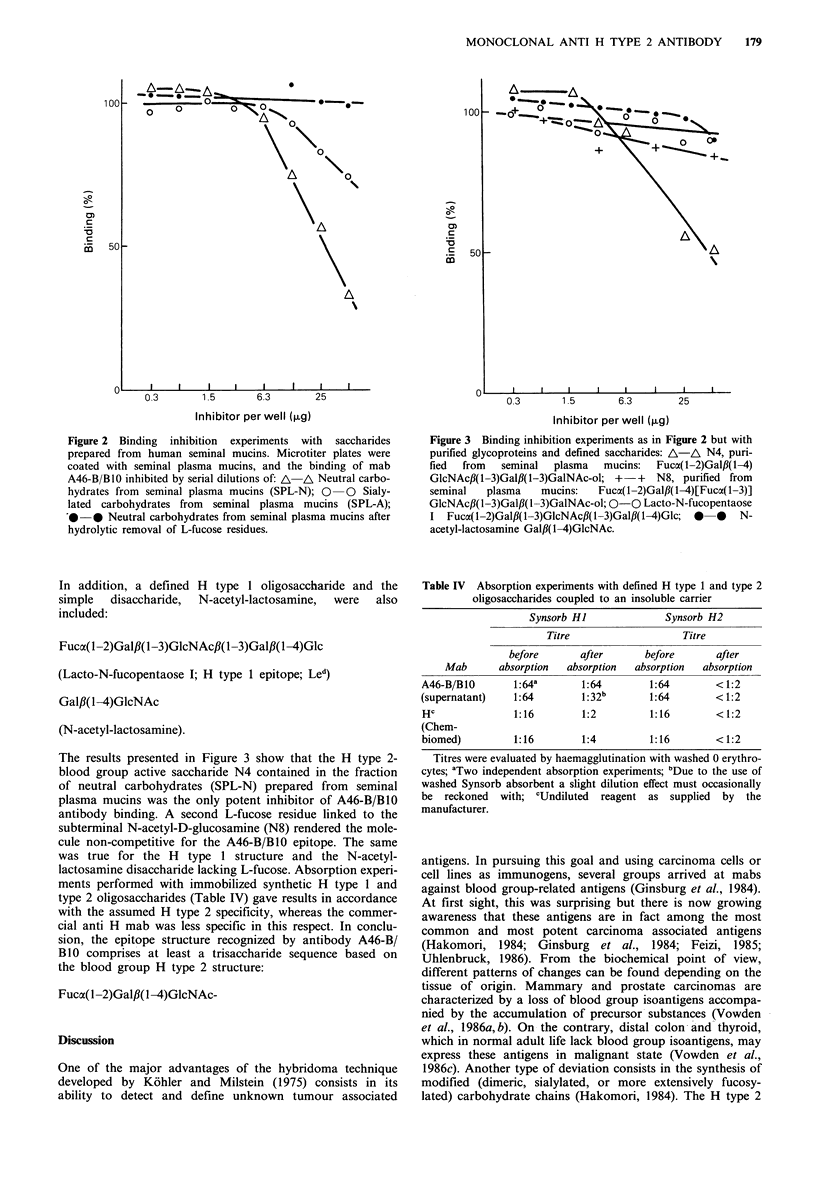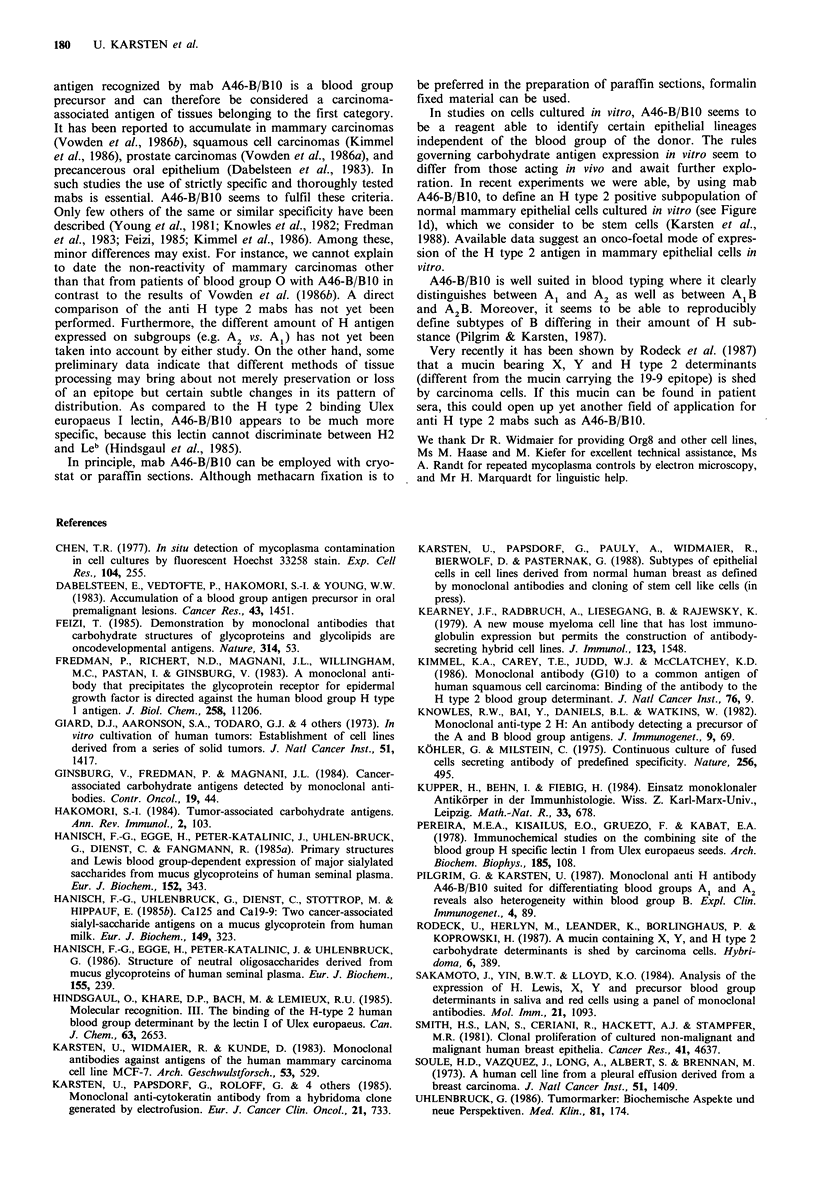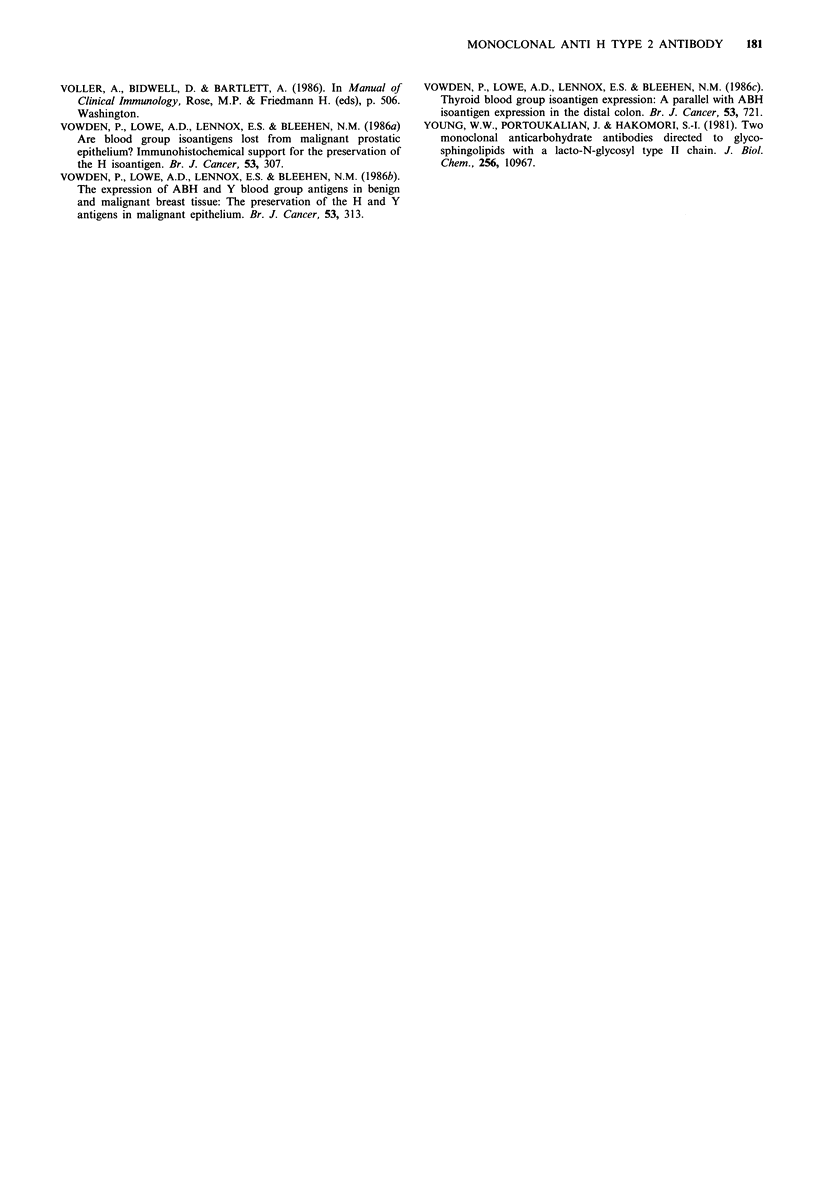# A new monoclonal antibody (A46-B/B10) highly specific for the blood group H type 2 epitope: generation, epitope analysis, serological and histological evaluation.

**DOI:** 10.1038/bjc.1988.187

**Published:** 1988-08

**Authors:** U. Karsten, G. Pilgrim, F. G. Hanisch, G. Uhlenbruck, M. Kasper, P. Stosiek, G. Papsdorf, G. Pasternak

**Affiliations:** Central Institute of Molecular Biology, Academy of Sciences of the GDR, Berlin-Buch.

## Abstract

**Images:**


*Br. J. Cancer* (1988), **58**, 176–181 © The Macmillan Press Ltd., 1988

### A new monoclonal antibody (A46-B/B10) highly specific for the blood group H type 2 epitope: Generation, epitope analysis, serological and histological evaluation

U. Karsten[1], G. Pilgrim[2], F.-G. Hanisch[3], G. Uhlenbruck[3], M. Kasper[4], P. Stosiek[4], G. Papsdorf[2] & G. Pasternak[1]

[1]*Central Institute of Molecular Biology, Academy of Sciences of the GDR, Berlin-Buch, GDR;* [2]*Central Institute of Cancer Research, Academy of Sciences of the GDR, Berlin-Buch, GDR;* [3]*Medical Clinic, University of Cologne, Cologne, FRG; and* [4]*Pathological Institute, District Hospital, Görlitz, GDR.*

**Summary** A monoclonal antibody recognizing the blood group H type 2 antigen has been obtained from a BALB/c mouse immunized with MCF-7 (human mammary carcinoma) cells. The specificity of this antibody (A46-B/B10, IgM, κ) has been identified by haemagglutination tests, immunohistochemistry, binding inhibition studies, and absorption experiments performed with synthetic oligosaccharides. The antibody is virtually nonreactive with H type 1 antigen or with closely related type 2 structures (e.g., Y antigen). A46-B/B10 strongly agglutinates human erythrocytes according to the amount of H substance expressed and can, therefore, easily discriminate between blood groups $A_1$ and $A_2$ as well as $A_1B$ and $A_2B$ ($A_1$ and $A_1B$ are not or only weakly agglutinated). In immunohistochemistry, this antibody seems to provide a highly specific reagent for a restricted number of carcinomas and epithelial lineages in tissue sections and *in vitro*.

Blood group-related carbohydrate antigens are important parts of membrane glycoproteins and glycolipids. They are thought to be involved in recognition processes. There is at present increasing interest in these substances because of accumulating evidence that they are characteristically modified during malignant transformation (Hakomori, 1984; Ginsburg *et al.*, 1984; Feizi, 1985; Uhlenbruck, 1986). Monoclonal antibodies (mabs) are essential tools for the analysis of these phenomena. We here describe a new mab specific for the H type 2 epitope and show its applicability in blood group serology as well as in immunohistochemistry.

### Materials and methods

### *Generation of hybridoma clone A46-B/B10*

Methodical details were as described in Karsten *et al.* (1983). Briefly, BALB/c mice were immunized with live cells of the human mammary carcinoma cell line MCF-7, derived from a patient of blood group O (Soule *et al.*, 1973). Spleen cells from one of these mice were fused with X63-Ag8.653 mouse myeloma cells (Kearney *et al.*, 1979), and supernatants from hybridomas growing in HAT medium were screened by means of indirect immunofluorescence tests performed with MCF-7 and A-204 (human rhabdomyosarcoma, Giard *et al.*, 1973) cells. Hybridoma A46-B/B10, which produced a mab reacting with a membrane antigen present on MCF-7 but absent on A-204 cells, was cloned repeatedly by limiting dilution on feeder cells, expanded and stored in liquid nitrogen. Cells of this clone were cultured in RPMI 1640 medium containing $5\times10^{-5}$ M 2-mercaptoethanol and 10% foetal calf or horse serum without antibiotics. (Horse serum, although well suited for hybridoma growth, disturbs some tests due to intrinsic anti carbohydrate antibodies). Hybridoma cells were checked for mycoplasma contamination by DNA staining (Chen, 1977) and electron microscopy with negative results. Ascites fluid was obtained in BALB/c mice conditioned by pristane and transplanted with 1 to $3\times10^6$ hybridoma cells. Isotype determination was done by immunodiffusion (heavy chain) and dot test (light chain) using specific antisera (Miles, Slough, UK).

Correspondence: U. Karsten, Zentralinstitut für Molekularbiologie der AdW, Robert-Rössle-Str. 10, DDR-1115 Berlin-Buch, GDR.
Received 6 August 1987; and in revised form, 22 March 1988.

### *Haematological methods*

Culture supernatants of A46-B/B10 (titres ranging from 1:16 to 1:64) were used in standard agglutination tests with washed erythrocytes resuspended in isotonic NaCl solution (for details see Pilgrim & Karsten, 1987).

### *Immunohistochemistry*

This was performed with either cell lines grown on multitest slides (Flow Labs., Bonn, FRG) and analyzed by indirect immunofluorescence or with cryostat sections cut from frozen tissue samples (4 μm; Frigocut, Reichert, Vienna, Austria) analyzed by means of a modified peroxidase anti peroxidase technique (Kupper *et al.*, 1984). Sections were counterstained with Mayer's haemalum. In some cases alternative techniques were employed, e.g. paraffin sections after formalin or methacarn fixation, or indirect immunoperoxidase staining. Peroxidase and FITC labelled second antibodies were from a commercial source (Staatliches Institut für Immunpräparate und Nährmedien, Berlin, GDR). Mabs with irrelevant specificity or culture medium alone served as negative controls.

### *Inhibition assays*

Quantitative analysis of antibody binding to seminal plasma mucins (4 μg ml$^{-1}$) and binding inhibition were performed as enzyme immunoassays on Immulon plates (Dynatech, Plochingen, FRG) according to Voller *et al.* (1976). Antibody A46-B/B10 produced as ascites was diluted 1:40 and mixed with serial two-fold dilutions of saccharides (starting from 50 μg well) or glycoprotein inhibitors (starting from 100 μg well). Lacto-N-fucopentaose I was purchased from BioCarb Chemicals, Lund, Sweden, and N-acetyllactosamine from Sigma, Munich, FRG. Glycoproteins from human secretions of serologically defined donors were enriched by hot phenol-saline extraction (Hanisch *et al.*, 1985*a*), and the mucins contained in these fractions were separated by exclusion chromatography on Sephacryl S 400 (Hanisch *et al.*, 1985*b*). Carbohydrate fractions and neutral saccharide alditols N4 and N8 with the primary structures Fucα(1–2) Galβ(1–4)GlcNAcβ(1–3)Galβ(1–3)GalNAc-ol and Fucα(1–2) Galβ (1–4)[Fucα(1–3)]GlcNAcβ (1–3) Galβ (1–3) GalNAc-ol, respectively, were isolated from mucins of human seminal fluid (Hanisch *et al.*, 1985*a*, 1986). Anti mouse IgG-phosphatase conjugate and *p*-nitrophenyl phosphate were from Sigma, Munich, FRG. Lectin inhibition was performed using a cell line expressing the A46-B/B10 antigen, H184A1 (mammary epithelial cells, Smith *et al.*, 1981). Cells were grown on multitest slides and stained in indirect immunofluorescence after preincubation with Ulex europaeus I lectin (Serva, Heidelberg, FRG) in serial dilutions (starting from 200 $\mu g\,ml^{-1}$) at 4°C for 1 h.

*Absorption with synthetic oligosaccharides*

Synthetic H type 1 and type 2 oligosaccharides bound to an inorganic carrier (Synsorb, Chembiomed, Edmonton, Canada) were washed with PBS, degassed, and incubated with A46-B/B10 supernatant at room temperature for 16 h with gentle shaking. Antibody titres before and after absorption were evaluated by haemagglutination with 0 erythrocytes. The absorption capacity of the Synsorb material had been estimated in preliminary experiments. Accordingly, 25 mg dry weight of immunosorbent was reacted with 100 $\mu l$ supernatant, titre 1:64. For comparison, an anti H mab commercially available from Chembiomed (Edmonton, Canada) was included. This mab had an original haemagglutination titre of 1:16.

### Results

The monoclonal antibody A46-B/B10 (IgM, $\kappa$) first occurred as an antibody strongly binding to a membrane antigen present on MCF-7 but absent on A-204 cells (Karsten *et al.*, 1983). Further examination against a panel of human cell lines (Table I) revealed the expression of this antigen on 4 out of 5 mammary carcinoma cell lines and on a mammary epithelial cell line derived from normal breast but transformed *in vitro*, H184A1. Furthermore, A46-B/B10 stained all of six carcinoma cell lines, whereas those derived from mesenchymal tissues (fibroblasts, sarcomas, lymphoid cells) were completey negative. Thus, at this stage, A46-B/B10 appeared to detect a carcinoma-associated antigen. Histological evaluation partly confirmed this conclusion. Carcinomas of breast and stomach were in a number of cases at least focally positive in contrast to their normal epithelial counterparts (Figure 1a,c). Among a number of mammary carcinomas, the expression of the A46-B/B10 antigen appeared to be blood group dependent (Table II). Moreover, this antigen was detected on certain normal tissues too, for instance, in the stratum spinosum and stratum lucidum of the skin, on acinar epithelial cells of pancreas (Figure 1b) and on epithelial cells of gall bladder and salivary glands. The reactivity of mab A46-B/B10 with endothelial cells of blood vessels (Figure 1c) and with erythrocytes of certain individuals again pointed towards a blood group-related antigen. This could then easily be confirmed by agglutination experiments with erythrocytes of knwon blood groups, the results of which are shown in Table III. Briefly, A46-B/B10 agglutinated strongly erythrocytes of blood groups O, $A_2$, and $A_2B$, but not, or only weakly, $A_1$ and $A_1B$. This pattern of reactivity suggested H antigen specificity. Among blood group B, both strongly and weakly reactive individuals were found. The agglutination results could be confirmed by absorption experiments with packed erythrocytes (not shown). Embryonal and adult blood samples gave similar agglutination patterns thus excluding Ii. MN could also be excluded by respective tests (not shown). Attempts to absorb A46-B/B10 activity with plasma or saliva from 0 secretors were negative (data not shown). Because saliva of secretors contains $Le^b$ and Y but not H type 2 antigen (Sakamoto *et al.*, 1984), this result was compatible with H type 2 specificity of mab A46-B/B10. This suggestion could be confirmed in further studies employing Ulex lectin and defined saccharides in binding inhibition experiments. Ulex europaeus I lectin preferentially binds H type 2 (Pereira *et al.*, 1978). In a cell line reactive with A46-B/B10 in immunofluorescence, H184A1 (Table I), binding of this mab, but not that of an unrelated mab, could be blocked by preincubation with the lectin in a dose-dependent manner. Pretreatment with 200 or 100 $\mu g\,ml^{-1}$ completely inhibited staining, whereas lower doses of the lectin were only partially effective. In a comparison of various human secretions, A46-B/B10 bound most strongly to glycoproteins from seminal fluid. These were accordingly used in binding inhibition studies. Binding was most effectively inhibited by gastric mucins from secretors or by neutral carbohydrates (SPL-N), which had been reductively cleaved from seminal plasma mucins (Figure 2). The corresponding sialic acid containing saccharide alditols from seminal mucins (SPL-A) were much less inhibitory. The inhibitory activity of neutral carbohydrates was abolished after chemical cleavage of L-fucose residues with mild acid. The involvement of L-fucose in the epitope structure recognized by mab A46-B/B10 was further examined with the following pure, chemically defined H type 2 related saccharides (Hanisch *et al.*, 1986):

Fucα(1–2)Galβ(1–4)GlcNAcβ(1–3)Galβ(1–3)GalNAc-ol

(N4; H type 2 epitope posing), and

Fucα(1–2)Galβ(1–4)[Fucα(1–3)]GlcNAcβ(1–3)

× Galβ(1–3)GalNAc-ol

(N8; Y epitope posing).

**Table I** Reactivity of mab A46-B/B10 with human cell lines

| *Cell line*[a] | *Tissue of origin* | *Donor blood group* | *Reactivity*[b] |
|---|---|---|---|
| MCF-7 | Mammary carcinoma | 0 | + |
| T47D | Mammary carcinoma | | + |
| BT-20 | Mammary carcinoma | | + |
| CAMA-1 | Mammary carcinoma | | + |
| MaTu | Mammary carcinoma | | – |
| H184A1 | Mammary epithelium | | + |
| Org8 | Mammary epithelium | $A_1$ | + |
| Hs578T | Carcinosarcoma of breast | | – |
| T-24 | Bladder carcinoma | | + |
| HT-29 | Colon carcinoma | A | (+) |
| LS-174T | Colon carcinoma | | (+) |
| HeLa-S | Epidermoid carcinoma of cervix | | (+) |
| A-431 | Epidermoid carcinoma of vulva | | (+) |
| Tu197 | Ovarian carcinoma | | (+) |
| A-204 | Rhabdomyosarcoma | | – |
| Tu256 | Osteogenic sarcoma | | – |
| Tu131 | Ewing sarcoma | | – |
| – | Skin fibroblasts | | – |
| HMy2 | Plasmacytoma | | – |
| Reh | Null cell ALL[c] | | – |
| Molt-4 | T-cell ALL | | – |
| SKW3 | T-cell leukaemia | | – |

[a]Sources, references, and cultivation conditions see Karsten *et al.*, 1983, 1985. Org8 is one out of a series of normal mammary epithelial cell lines described in Karsten *et al.* (1988); [b]Reactiviity indicated as follows: – = no staining; (+) = weak staining; + = strong staining; [c]ALL = acute lymphoblastic leukaemia.

**Table II** Staining characteristics of mab A46-B/B10 with cryostat sections of mammary carcinomas

| *Blood group* | *No.* | ∅ | + | ++ | +++ |
|---|---|---|---|---|---|
| A | 8 | 8 | – | – | – |
| B | 3 | 3 | – | – | – |
| O | 6 | 2 | 2 | 1 | 1 |
| Total | 17 | 13 | 2 | 1 | 1 |

Numbers indicate specimens belonging to each group. Staining intensity is given as follows: ∅ = negative (no staining); + = less than 10%, ++ = 10 to 90%; +++ = more than 90% of carcinoma cells stained.

**Figure 1** Examples of cells and tissues expressing the A46-B/B10 antigen (H type 2). Bar = 20 $\mu$m. (a) Cryostat section of mammary carcinoma. Carcinoma cells focally positive, stromal cells negative; (b) Cryostat section of normal pancreas. Acinar epithelial cells (exocrine pancreas = ep) positive, islet cells (i) negative; (c) Methacarn fixed paraffin section from normal breast (reduction mammoplasty, blood group O). Endothelial cells positive, epithelial and stromal cells negative. (a)–(c) Peroxidase technique, counterstained with haemalum. In (c) a strong blue filter was applied in order to reduce the optical dominance of counterstained nuclei. (d) Cell line Org8 (mammary epithelial cell line derived from a patient blood group $A_1$, undergoing reduction mammoplasty). Indirect immunofluorescence technique.

**Table III** Agglutination of a total of 1138 samples of known blood groups with mab A46-B/B10

| *Agglutination strength*[a] | *Blood group* | | | | | |
|---|---|---|---|---|---|---|
| | *O* | $A_2$ | $A_2B$ | $A_1$ | $A_1B$ | *B* |
| + + + + | 181[b] (87.4)[c] | 69 (71.9) | 19 (41.3) | 3 (1.2) | 0 (0.0) | 113 (26.1) |
| + + + | 26 (12.6) | 23 (23.9) | 23 (50.0) | 18 (7.2) | 0 (0.0) | 151 (34.9) |
| + + | 0 (0.0) | 4 (4.2) | 4 (8.7) | 39 (15.6) | 18 (17.0) | 102 (23.6) |
| + | 0 (0.0) | 0 (0.0) | 0 (0.0) | 89 (35.6) | 27 (25.5) | 56 (12.9) |
| ∅ | 0 (0.0) | 0 (0.0) | 0 (0.0) | 101 (40.4) | 61 (57.5) | 11 (2.5) |
| Total[d] | 207 | 96 | 46 | 250 | 106 | 433 |

[a]Agglutination strength scored as follows: 0 = negative; + = very small agglutinates; + + = small but definite agglutinates; + + + = several clots in clear fluid; + + + + = clot in clear fluid; [b]Numbers of samples examined; [c]Per cent of samples per blood group reacting according to indicated the agglutination strength; [d]These numbers do not reflect the relative proportions of blood groups in the population.

Figure 2 Binding inhibition experiments with saccharides prepared from human seminal mucins. Microtiter plates were coated with seminal plasma mucins, and the binding of mab A46-B/B10 inhibited by serial dilutions of: △—△ Neutral carbohydrates from seminal plasma mucins (SPL-N); ○—○ Sialylated carbohydrates from seminal plasma mucins (SPL-A); ●—● Neutral carbohydrates from seminal plasma mucins after hydrolytic removal of L-fucose residues.

Figure 3 Binding inhibition experiments as in Figure 2 but with purified glycoproteins and defined saccharides: △—△ N4, purified from seminal plasma mucins: Fucα(1–2)Galβ(1–4) GlcNAcβ(1–3)Galβ(1–3)GalNAc-ol; +—+ N8, purified from seminal plasma mucins: Fucα(1–2)Galβ(1–4)[Fucα(1–3)] GlcNAcβ(1–3)Galβ(1–3)GalNAc-ol; ○—○ Lacto-N-fucopentaose I Fucα(1–2)Galβ(1–3)GlcNAcβ(1–3)Galβ(1–4)Glc; ●—● N-acetyl-lactosamine Galβ(1–4)GlcNAc.

In addition, a defined H type 1 oligosaccharide and the simple disaccharide, N-acetyl-lactosamine, were also included:

Fucα(1–2)Galβ(1–3)GlcNAcβ(1–3)Galβ(1–4)Glc

(Lacto-N-fucopentaose I; H type 1 epitope; Le[d])

Galβ(1–4)GlcNAc

(N-acetyl-lactosamine).

The results presented in Figure 3 show that the H type 2-blood group active saccharide N4 contained in the fraction of neutral carbohydrates (SPL-N) prepared from seminal plasma mucins was the only potent inhibitor of A46-B/B10 antibody binding. A second L-fucose residue linked to the subterminal N-acetyl-D-glucosamine (N8) rendered the molecule non-competitive for the A46-B/B10 epitope. The same was true for the H type 1 structure and the N-acetyl-lactosamine disaccharide lacking L-fucose. Absorption experiments performed with immobilized synthetic H type 1 and type 2 oligosaccharides (Table IV) gave results in accordance with the assumed H type 2 specificity, whereas the commercial anti H mab was less specific in this respect. In conclusion, the epitope structure recognized by antibody A46-B/B10 comprises at least a trisaccharide sequence based on the blood group H type 2 structure:

Fucα(1–2)Galβ(1–4)GlcNAc-

Table IV Absorption experiments with defined H type 1 and type 2 oligosaccharides coupled to an insoluble carrier

| | *Synsorb H1* | | *Synsorb H2* | |
|---|---|---|---|---|
| | *Titre* | | *Titre* | |
| *Mab* | *before absorption* | *after absorption* | *before absorption* | *after absorption* |
| A46-B/B10 | 1:64[a] | 1:64 | 1:64 | <1:2 |
| (supernatant) | 1:64 | 1:32[b] | 1:64 | <1:2 |
| H[c] | 1:16 | 1:2 | 1:16 | <1:2 |
| (Chem-biomed) | 1:16 | 1:4 | 1:16 | <1:2 |

Titres were evaluated by haemagglutination with washed 0 erythrocytes; [a]Two independent absorption experiments; [b]Due to the use of washed Synsorb absorbent a slight dilution effect must occasionally be reckoned with; [c]Undiluted reagent as supplied by the manufacturer.

### Discussion

One of the major advantages of the hybridoma technique developed by Köhler and Milstein (1975) consists in its ability to detect and define unknown tumour associated antigens. In pursuing this goal and using carcinoma cells or cell lines as immunogens, several groups arrived at mabs against blood group-related antigens (Ginsburg *et al.*, 1984). At first sight, this was surprising but there is now growing awareness that these antigens are in fact among the most common and most potent carcinoma associated antigens (Hakomori, 1984; Ginsburg *et al.*, 1984; Feizi, 1985; Uhlenbruck, 1986). From the biochemical point of view, different patterns of changes can be found depending on the tissue of origin. Mammary and prostate carcinomas are characterized by a loss of blood group isoantigens accompanied by the accumulation of precursor substances (Vowden *et al.*, 1986*a*, *b*). On the contrary, distal colon and thyroid, which in normal adult life lack blood group isoantigens, may express these antigens in malignant state (Vowden *et al.*, 1986*c*). Another type of deviation consists in the synthesis of modified (dimeric, sialylated, or more extensively fucosylated) carbohydrate chains (Hakomori, 1984). The H type 2

antigen recognized by mab A46-B/B10 is a blood group precursor and can therefore be considered a carcinoma-associated antigen of tissues belonging to the first category. It has been reported to accumulate in mammary carcinomas (Vowden *et al.*, 1986*b*), squamous cell carcinomas (Kimmel *et al.*, 1986), prostate carcinomas (Vowden *et al.*, 1986*a*), and precancerous oral epithelium (Dabelsteen *et al.*, 1983). In such studies the use of strictly specific and thoroughly tested mabs is essential. A46-B/B10 seems to fulfil these criteria. Only few others of the same or similar specificity have been described (Young *et al.*, 1981; Knowles *et al.*, 1982; Fredman *et al.*, 1983; Feizi, 1985; Kimmel *et al.*, 1986). Among these, minor differences may exist. For instance, we cannot explain to date the non-reactivity of mammary carcinomas other than that from patients of blood group O with A46-B/B10 in contrast to the results of Vowden *et al.* (1986*b*). A direct comparison of the anti H type 2 mabs has not yet been performed. Furthermore, the different amount of H antigen expressed on subgroups (e.g. $A_2$ *vs.* $A_1$) has not yet been taken into account by either study. On the other hand, some preliminary data indicate that different methods of tissue processing may bring about not merely preservation or loss of an epitope but certain subtle changes in its pattern of distribution. As compared to the H type 2 binding Ulex europaeus I lectin, A46-B/B10 appears to be much more specific, because this lectin cannot discriminate between H2 and $Le^b$ (Hindsgaul *et al.*, 1985).

In principle, mab A46-B/B10 can be employed with cryostat or paraffin sections. Although methacarn fixation is to be preferred in the preparation of paraffin sections, formalin fixed material can be used.

In studies on cells cultured *in vitro*, A46-B/B10 seems to be a reagent able to identify certain epithelial lineages independent of the blood group of the donor. The rules governing carbohydrate antigen expression *in vitro* seem to differ from those acting *in vivo* and await further exploration. In recent experiments we were able, by using mab A46-B/B10, to define an H type 2 positive subpopulation of normal mammary epithelial cells cultured *in vitro* (see Figure 1d), which we consider to be stem cells (Karsten *et al.*, 1988). Available data suggest an onco-foetal mode of expression of the H type 2 antigen in mammary epithelial cells *in vitro*.

A46-B/B10 is well suited in blood typing where it clearly distinguishes between $A_1$ and $A_2$ as well as between $A_1B$ and $A_2B$. Moreover, it seems to be able to reproducibly define subtypes of B differing in their amount of H substance (Pilgrim & Karsten, 1987).

Very recently it has been shown by Rodeck *et al.* (1987) that a mucin bearing X, Y and H type 2 determinants (different from the mucin carrying the 19-9 epitope) is shed by carcinoma cells. If this mucin can be found in patient sera, this could open up yet another field of application for anti H type 2 mabs such as A46-B/B10.

We thank Dr R. Widmaier for providing Org8 and other cell lines, Ms M. Haase and M. Kiefer for excellent technical assistance, Ms A. Randt for repeated mycoplasma controls by electron microscopy, and Mr H. Marquardt for linguistic help.

### References

CHEN, T.R. (1977). *In situ* detection of mycoplasma contamination in cell cultures by fluorescent Hoechst 33258 stain. *Exp. Cell Res.*, **104**, 255.

DABELSTEEN, E., VEDTOFTE, P., HAKOMORI, S.-I. & YOUNG, W.W. (1983). Accumulation of a blood group antigen precursor in oral premalignant lesions. *Cancer Res.*, **43**, 1451.

FEIZI, T. (1985). Demonstration by monoclonal antibodies that carbohydrate structures of glycoproteins and glycolipids are oncodevelopmental antigens. *Nature*, **314**, 53.

FREDMAN, P., RICHERT, N.D., MAGNANI, J.L., WILLINGHAM, M.C., PASTAN, I. & GINSBURG, V. (1983). A monoclonal antibody that precipitates the glycoprotein receptor for epidermal growth factor is directed against the human blood group H type 1 antigen. *J. Biol. Chem.*, **258**, 11206.

GIARD, D.J., AARONSON, S.A., TODARO, G.J. & 4 others (1973). *In vitro* cultivation of human tumors: Establishment of cell lines derived from a series of solid tumors. *J. Natl Cancer Inst.*, **51**, 1417.

GINSBURG, V., FREDMAN, P. & MAGNANI, J.L. (1984). Cancer-associated carbohydrate antigens detected by monoclonal antibodies. *Contr. Oncol.*, **19**, 44.

HAKOMORI, S.-I. (1984). Tumor-associated carbohydrate antigens. *Ann. Rev. Immunol.*, **2**, 103.

HANISCH, F.-G., EGGE, H., PETER-KATALINIC, J., UHLEN-BRUCK, G., DIENST, C. & FANGMANN, R. (1985*a*). Primary structures and Lewis blood group-dependent expression of major sialylated saccharides from mucus glycoproteins of human seminal plasma. *Eur. J. Biochem.*, **152**, 343.

HANISCH, F.-G., UHLENBRUCK, G., DIENST, C., STOTTROP, M. & HIPPAUF, E. (1985*b*). Ca125 and Ca19-9: Two cancer-associated sialyl-saccharide antigens on a mucus glycoprotein from human milk. *Eur. J. Biochem.*, **149**, 323.

HANISCH, F.-G., EGGE, H., PETER-KATALINIC, J. & UHLENBRUCK, G. (1986). Structure of neutral oligosaccharides derived from mucus glycoproteins of human seminal plasma. *Eur. J. Biochem.*, **155**, 239.

HINDSGAUL, O., KHARE, D.P., BACH, M. & LEMIEUX, R.U. (1985). Molecular recognition. III. The binding of the H-type 2 human blood group determinant by the lectin I of Ulex europaeus. *Can. J. Chem.*, **63**, 2653.

KARSTEN, U., WIDMAIER, R. & KUNDE, D. (1983). Monoclonal antibodies against antigens of the human mammary carcinoma cell line MCF-7. *Arch. Geschwulstforsch.*, **53**, 529.

KARSTEN, U., PAPSDORF, G., ROLOFF, G. & 4 others (1985). Monoclonal anti-cytokeratin antibody from a hybridoma clone generated by electrofusion. *Eur. J. Cancer Clin. Oncol.*, **21**, 733.

KARSTEN, U., PAPSDORF, G., PAULY, A., WIDMAIER, R., BIERWOLF, D. & PASTERNAK, G. (1988). Subtypes of epithelial cells in cell lines derived from normal human breast as defined by monoclonal antibodies and cloning of stem cell like cells (in press).

KEARNEY, J.F., RADBRUCH, A., LIESEGANG, B. & RAJEWSKY, K. (1979). A new mouse myeloma cell line that has lost immunoglobulin expression but permits the construction of antibody-secreting hybrid cell lines. *J. Immunol.*, **123**, 1548.

KIMMEL, K.A., CAREY, T.E., JUDD, W.J. & MCCLATCHEY, K.D. (1986). Monoclonal antibody (G10) to a common antigen of human squamous cell carcinoma: Binding of the antibody to the H type 2 blood group determinant. *J. Natl Cancer Inst.*, **76**, 9.

KNOWLES, R.W., BAI, Y., DANIELS, B.L. & WATKINS, W. (1982). Monoclonal anti-type 2 H: An antibody detecting a precursor of the A and B blood group antigens. *J. Immunogenet.*, **9**, 69.

KÖHLER, G. & MILSTEIN, C. (1975). Continuous culture of fused cells secreting antibody of predefined specificity. *Nature*, **256**, 495.

KUPPER, H., BEHN, I. & FIEBIG, H. (1984). Einsatz monoklonaler Antikörper in der Immunhistologie. Wiss. Z. Karl-Marx-Univ., Leipzig. *Math.-Nat. R.*, **33**, 678.

PEREIRA, M.E.A., KISAILUS, E.O., GRUEZO, F. & KABAT, E.A. (1978). Immunochemical studies on the combining site of the blood group H specific lectin 1 from Ulex europaeus seeds. *Arch. Biochem. Biophys.*, **185**, 108.

PILGRIM, G. & KARSTEN, U. (1987). Monoclonal anti H antibody A46-B/B10 suited for differentiating blood groups $A_1$ and $A_2$ reveals also heterogeneity within blood group B. *Expl. Clin. Immunogenet.*, **4**, 89.

RODECK, U., HERLYN, M., LEANDER, K., BORLINGHAUS, P. & KOPROWSKI, H. (1987). A mucin containing X, Y, and H type 2 carbohydrate determinants is shed by carcinoma cells. *Hybridoma*, **6**, 389.

SAKAMOTO, J., YIN, B.W.T. & LLOYD, K.O. (1984). Analysis of the expression of H. Lewis, X, Y and precursor blood group determinants in saliva and red cells using a panel of monoclonal antibodies. *Mol. Imm.*, **21**, 1093.

SMITH, H.S., LAN, S., CERIANI, R., HACKETT, A.J. & STAMPFER, M.R. (1981). Clonal proliferation of cultured non-malignant and malignant human breast epithelia. *Cancer Res.*, **41**, 4637.

SOULE, H.D., VAZQUEZ, J., LONG, A., ALBERT, S. & BRENNAN, M. (1973). A human cell line from a pleural effusion derived from a breast carcinoma. *J. Natl Cancer Inst.*, **51**, 1409.

UHLENBRUCK, G. (1986). Tumormarker: Biochemische Aspekte und neue Perspektiven. *Med. Klin.*, **81**, 174.

VOLLER, A., BIDWELL, D. & BARTLETT, A. (1986). In *Manual of Clinical Immunology*, Rose, M.P. & Friedmann H. (eds), p. 506. Washington.

VOWDEN, P., LOWE, A.D., LENNOX, E.S. & BLEEHEN, N.M. (1986*a*) Are blood group isoantigens lost from malignant prostatic epithelium? Immunohistochemical support for the preservation of the H isoantigen. *Br. J. Cancer*, **53**, 307.

VOWDEN, P., LOWE, A.D., LENNOX, E.S. & BLEEHEN, N.M. (1986*b*). The expression of ABH and Y blood group antigens in benign and malignant breast tissue: The preservation of the H and Y antigens in malignant epithelium. *Br. J. Cancer*, **53**, 313.

VOWDEN, P., LOWE, A.D., LENNOX, E.S. & BLEEHEN, N.M. (1986*c*). Thyroid blood group isoantigen expression: A parallel with ABH isoantigen expression in the distal colon. *Br. J. Cancer*, **53**, 721.

YOUNG, W.W., PORTOUKALIAN, J. & HAKOMORI, S.-I. (1981). Two monoclonal anticarbohydrate antibodies directed to glycosphingolipids with a lacto-N-glycosyl type II chain. *J. Biol. Chem.*, **256**, 10967.